# PML::RARα+ myeloid cells display metabolic alterations that can be targeted to treat resistant/relapse acute promyelocytic leukemias

**DOI:** 10.1038/s41375-025-02738-9

**Published:** 2025-09-10

**Authors:** Alessandra Zaza, Giuseppe Zardo, Cristina Banella, Sara Tucci, Elisabetta de Marinis, Martina Gentile, Serena Travaglini, Mariadomenica Divona, Tiziana Ottone, Germana Castelli, Anna Maria Cerio, Daniela F. Angelini, Isabella Faraoni, Raffaele Palmieri, Paquale Niscola, Emanuele Ammatuna, Adriano Venditti, Clara Nervi, Maria Teresa Voso, Gianfranco Catalano, Nelida Ines Noguera

**Affiliations:** 1 I.R.C.C.S Santa Lucia Foundation, Via del Fosso di Fiorano, Rome, Italy; 2 Department of Medical and Surgical Sciences and Biotechnologies, University of Roma La Sapienza, Rome, Italy; 3 Department of Experimental Medicine, Sapienza University, Rome, Italy; 4 Department of Health Sciences, University of Florence and Meyer Children’s University Hospital, Florence, Italy; 5 Pharmacy, Faculty of Medicine and Medical Centre, University of Freiburg, Freiburg, Germany; 6 Department of Experimental Medicine, Tor Vergata University, Rome, Italy; 7 Department of Biomedicine and Prevention, Tor Vergata University, Rome, Italy; 8 Department of Hematology, Oncology and Molecular Medicine, Istituto Superiore di Sanità, 00161 Rome, Italy; 9 Neuroimmunology and Flow Cytometry Units, Santa Lucia Foundation, IRCCS, Rome, Italy; 10 Department of Systems Medicine, Tor Vergata University, Rome, Italy; 11 Hematology Unit, S. Eugenio Hospital (ASL Roma 2), Rome, Italy; 12 Department of Hematology, University of Groningen, University Medical Center Groningen, Groningen, The Netherlands

**Keywords:** Acute myeloid leukaemia, Preclinical research

## Abstract

At present there is no metabolic characterization of acute promyelocytic leukemia (APL). Pathognomonic of APL, PML::RARα fusion protein rewires metabolic pathways to feed anabolic tumor cell’s growth. All-trans retinoic acid (ATRA) and arsenic trioxide (ATO)-based therapies render APL the most curable subtype of AML, yet approximately 1% of cases are resistant and 5% relapse. We characterized the metabolic peculiarity and fuel requirement of PML::RARα expressing cells, to identify new targets for tailored therapies in resistant or relapsed APL patients. We analyzed cell metabolism in primary samples from seven APL patients, comparing them with normal CD34+ cells differentiated to promyelocyte and granulocyte, and different PML::RARα expressing cell lines. We show that the PML::RARα oncoprotein inhibits glycolysis, promotes tricarboxylic acid cycle (TCA), and favors long chain fatty acids (LCFA) catabolism. Targeting CD36 function, that promotes the cellular uptake of fatty acids to feed oxidative phosphorylation (OXPHOS), effectively restores sensitivity to ATO in NB4 ATO-resistant clones. Notably, our data demonstrate that glycolytic impairment via AKT inhibition by PML::RARα renders APL cells reliant on OXPHOS. This dependency confers high sensitivity to the VTX-AZA combination, suggesting the therapeutic efficacy of targeted combination treatment in resistant or relapsed APLs.

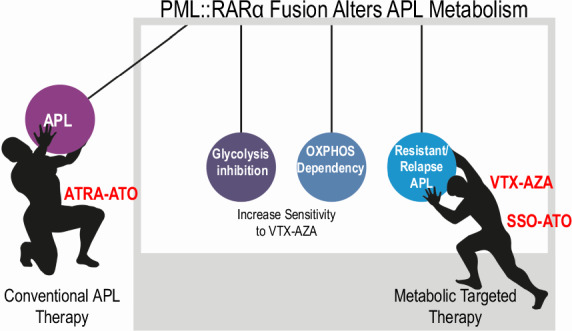

## Introduction

APL is characterized by the t(15;17) chromosomal translocation creating the *PML::RAR*α fusion gene, which is central in the pathogenesis of APL [1–4]. PML::RARα fusion oncoprotein impairs the formation of functional PML nuclear bodies, acts as a transcriptional repressor antagonizing myeloid differentiation, alters DNA repair and oxidoreductive state of the cell, promotes self-renewal of APL initiating cells and leukemia progression [5–8]. The epidemiological and clinical peculiarities of the disease: seasonality, excess of high body mass index (BMI) patients [9, 10], sudden onset, make for a complex biological background yet to be studied. All-trans retinoic acid (ATRA) and arsenic trioxide (ATO)-based therapies have led to a marked improvement in patient survival rates, and APL is now the most curable subtype of AML when diagnosed and treated promptly [11, 12].

ATRA binds to the PML::RARα fusion protein, leading to transcriptional activation of RARα target genes and differentiation of APL blasts, whereas ATO binds to the B2 domain of PML and induces PML::RARα degradation causing apoptosis [13, 14].

Approximately 1% of APL cases are resistant to ATRA/ATO therapy and 5% APL cases relapse, eventually becoming resistant to treatment [15]. In resistant patients, hematologic stem cell transplantation (allo-HSCT) is the only curative approach [3, 11]. Point mutations in the ATRA and ATO binding domains are found in 27 to 45% of resistant APL cases [16–18], and occasionally mutations in other proteins (e.g. PRMT5 involved in ATO resistance) [19]. Since these alterations do not cover the generality of the cases, it is conceivable that additional molecular mechanisms contribute to resistance.

Given that APL relapse is mainly due to therapy resistance, tailored treatments with new drugs that synergize with, or re-sensitize cells to standard treatment are of urgent need.

A wide metabolic reprogramming is necessary to fulfill the anabolic and energy-producing needs that enable cancer cell growth. Leukemia-associated cellular reprogramming in hematopoietic stem cells (HSCs) subverts their physiological metabolic pathways [20, 21]. The metabolic heterogeneity at leukemia onset and the metabolic clonal evolution driven by therapy are essential insights that remain unclear. PML::RARα directly and indirectly impacts leukemic cells’ metabolic pathways. A high-throughput transcriptional profile analysis and metabolic characterization of NB4 cells, a well-established APL experimental cell model, revealed a considerable reprogramming of genes involved in cancer metabolism [22]. RNA-sequencing analysis on 42 APL patients’ samples, individuated two APL metabolic subtypes showing distinct transcriptional regulation and prognosis [23]. Nevertheless, at present, there is no experimental evidence of a functional characterization of APL from a metabolic point of view.

Aberrant metabolism is not only central for leukemia cells proliferation and survival but also allows the emergence of cellular subclones carrying new phenotypes that have been found significant for disease evolution, response and resistance to therapy [24]. Targeting the energetic supply or the mitochondrial electron transport chain (ETC) itself may provide new therapeutic opportunities to overcome APL relapse or resistance to therapy. The individuation of common metabolic peculiarities in APL cells in general, and in therapy resistant clones, will provide the mean to identify new therapeutic targets, facilitating the choice of specific drugs and the development of active and safe agents to be tested in clinical trials.

## Methods

### Patient samples and controls

Bone marrow (BM) mononuclear cells (MNC) were collected from 7 patients with APL diagnosis at onset, admitted at the Department of Hematology of the University of Rome Tor Vergata. *PML::RAR*α presence was confirmed in all cases by RT-PCR. BM-infiltration by leukemic blasts was >60% in all patients. BM-MNCs isolated from healthy individuals were used as controls. CD34+ hematopoietic progenitors were isolated from the cord blood (CB), and used as normal controls. Written informed consent was obtained from all subjects according to institutional guidelines and the Declaration of Helsinki. The study was approved by the ethical committee of the University of Rome Tor Vergata, experimentation register number 24.24 CET2 ptv. Details on CD34+ hematopoietic progenitors in vitro cultures and AML cell lines are in the Supplementary Methods.

### Cell viability assay

- The ATP-based assay CellTiter-Glo® Viability Assay (Promega®, Madison, USA) performed according to manufacturer’s instructions.
- MTS assay A CellTiter 96® AQueous One Solution Cell Proliferation Assay performed according to manufacturer’s instructions. (Supplementary Methods).

Reagents used are reported in Supplementary Table S3.

### Seahorse assays

Glycolytic and mitochondrial activities were evaluated using a Seahorse Bioscience XFe96 analyzer in combination with the Seahorse Bioscience XF Cell Mito Stress Test and the Bioscience XF Cell Glycolysis Stress Test (Agilent Technologies), respectively as reported [22, 25] (Supplementary Methods).

### Western blot analysis

Western blot analysis performed as previously described [26]. The primary antibodies used are reported in Supplementary Table S4.

### Quantitative real-time expression analysis

The expression levels of *SLC22A16* and *PDHA1* mRNAs were evaluated by Q-RT-PCR with the LightCycler® 480 Real-Time PCR-System (Roche, Germany) [27]. The primer sequences are listed in Supplementary Table S5.

### Metabolic assays

The Analysis of amino acids, acylcarnitines and TCA cycle intermediates are described in Supplementary Methods.

## Results

### PML::RARα inhibits glycolysis via AKT degradation

We profiled metabolism and nutrients requirements in primary samples from 7 APL patients (molecular and clinical characteristics are shown in Supplementary Table-S1). The metabolic peculiarities of APL blasts were compared to those of human hematopoietic progenitors/precursors isolated from healthy donors’ bone marrow (NBM) or from cord blood (CB) CD34+ cells (EP/P), undergoing sequential stages of granulocytic differentiation/maturation in culture. Neutrophilic precursors were collected on day 7 (N7, mostly promyelocytes) or day 13 of culture (N13, mostly granulocytes), as previously reported [28, 29].

**Fig. 1:**
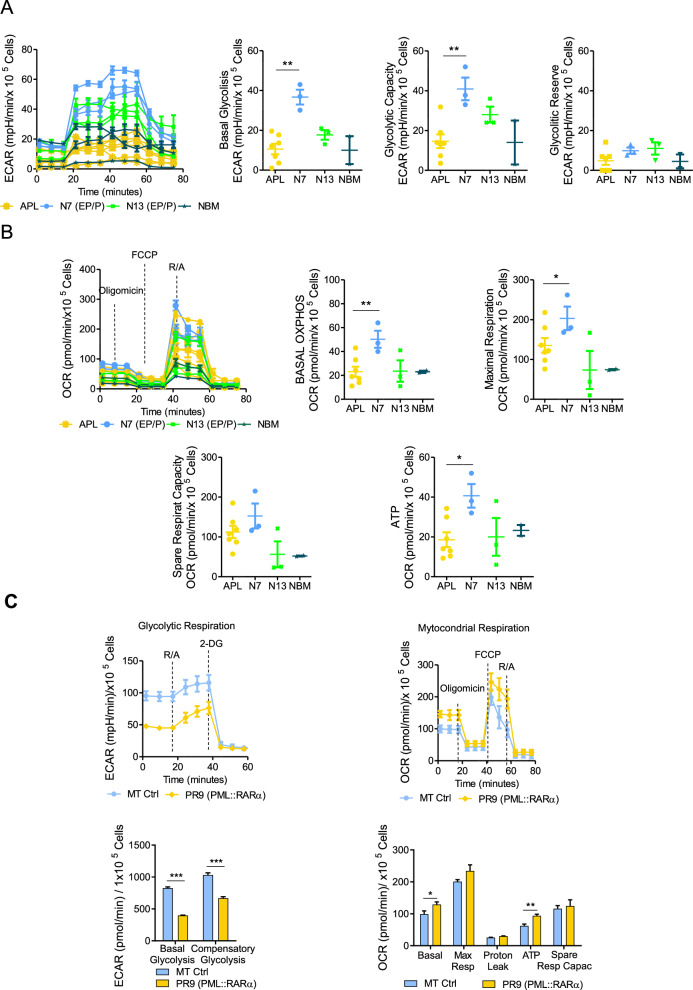
Metabolic characterization of normal hematopoietic precursors/progenitors, primary APL blasts and myeloid cell lines expressing the PML::RARα oncoprotein. **A** Extracellular acidification rates (ECAR), indicative of basal glycolysis, glycolytic reserve and glycolytic capacity, and **B** oxygen consumption rates (OCR), indicative of basal respiration, maximal respiration, spare respiratory capacity and mitochondrial ATP production, were measured in primary blasts from APL patients (*n* = 7), hematopoietic precursors /progenitors (EP/P) at day 7 (N7, mostly promyelocytes, *n* = 3), and day 13 (N13 mostly neutrophil granulocytes, *n* = 3), and in normal bone marrow (NBM, *n* = 2). Molecular and clinical characterization of APL primary blast is shown in Supplementary Table-S1 (N°1 to 7). Statistical analysis was performed using the non-parametric Mann Whitney t test; (**C left**) Profile of the glycolytic respiration and (**C right**) mitochondrial respiration in MT cells (Ctrl) and in PR9 cells (PML::RARα + ) treated with 100 µM ZnSO_4_ for 24 h. Experiments were performed in two independent biological replicates. Statistical analysis was performed using the Student’s *t*-test. * *p* ≤ 0.05, **p ≤ 0.005, ***p ≤ 0.0005.

By evaluating the extracellular acidification rate (ECAR), we found that basal glycolysis, glycolytic capacity and glycolytic reserve levels in primary APL blasts were comparable to those observed in EP/P-N13 and NBM cells. However, APL blasts showed lower basal glycolysis (*p* = 0.001), lower glycolytic capacity (*p* = 0.01), and comparable glycolytic reserve to normal promyelocytes (EP/P-N7) (Fig. 1A and Supplementary Table-S2).

APL blasts presented lower levels of OCR (oxidative phosphorylation/OXPHOS), mitochondrial respiration rate than normal promyelocytes (EP/P-N7) (*p* = 0.003), and comparable values to normal granulocytes (EP/P-N13) and NBM. The spare respiratory capacity in APL blasts was lower with respect to normal promyelocytes (EP/P-N7) (*p* = 0.27), but higher than in granulocytes (EP/P-N13) and NBM (Fig. 1B and Supplementary Table-S2).

Our results indicate that, in contrast to normal promyelocyte (EP/P-7), APL blasts may be particularly vulnerable to OXPHOS inhibition due to their lower glycolytic capacity.

To investigate further the metabolic changes occurring in APL blasts and to define the role of PML::RARα in these events, we used the PR9 cells (U937 cells carrying the PML::RARα cDNA under a ZnSO_4_-inducible promoter) and control (MT) cells (U937 cells bearing an empty vector). PML::RARα expression in PR9 cells was associated to a significant inhibition of basal glycolysis (*p* < 0.0001) and compensatory glycolysis (*p* < 0.0001), compared to MT control cells (Fig. 1C left and Supplementary Table-S3). These results are in line with the reduced glycolytic levels observed in primary APL blasts when compared with normal promyelocytes. However, an increased mitochondrial respiration was also measured in PR9 cells (PML::RARα + ) (Fig. 1C right and Supplementary Table-S3).

**Fig. 2:**
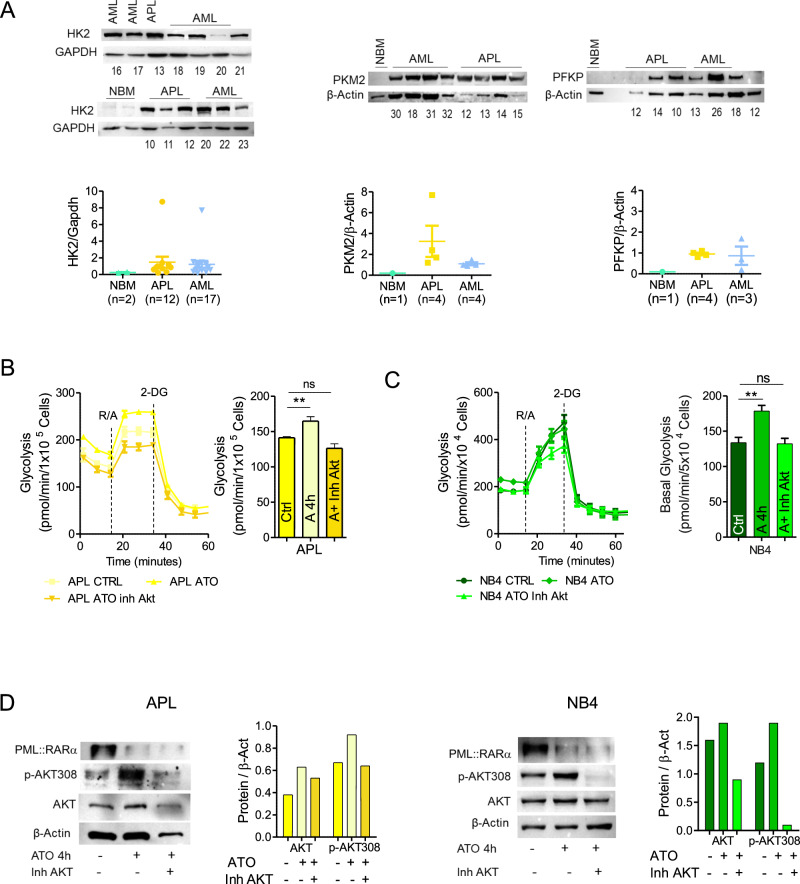
PML-RARα inhibits glycolysis via AKT degradation. **A** HK2, PKM2 and PFKP protein expression were analyzed in primary blasts obtained from APL and AML patients, and normal bone marrow cells (NBM). The numbers at the bottom of the western blot identify the patients described in Supplementary Table-S1. **B** Effects of 1 µM ATO treatment for 4 h in combination or not with 1 µM AKT inhibitor (Inhibitor VIII) for 30 min on the glycolytic activity in Fresh blasts from one APL patient (n° 8, Supplementary Table-S1) and **C** NB4 cells. Histograms represent basal glycolysis measured with the XF Glycolytic rate assay from two independent replicates. **D** Western blot detecting PML::RARα, AKT and p-AKT308 protein expression levels in (**left**) fresh blasts from an APL patient (n° 8, Supplementary Table-S1), (**right**) NB4 cells undergoing the treatments reported above. Protein values were analyzed by densitometric scanning and reported in the histogram after normalization with β-actin. Statistical analysis was performed using the Student’s t-test, ** *p* ≤ 0.005.

Although the expression of PML::RARα in PR9 cells inhibited glycolysis, the protein levels of key glycolytic enzymes, such as the Hexokinase 2 (HK2), the isoenzyme of Pyruvate kinase (PKM2) and Phosphofructokinase (PFKP), are not decreased following the induction of PML::RARα expression (Supplementary Fig.-S1A). The expression levels of these enzymes are significantly higher in APL and AML blasts when compared to NBM cells (Fig. 2A), indicating that a different mechanism leads to the APL glycolytic inefficiency.

We previously demonstrated that HSP90 expression is inhibited by PML::RARα, leading to degradation of AKT, an important regulator of cell growth, survival and metabolism [30–32]. To investigate the role of AKT in glycolysis inhibition, fresh APL blasts were treated with ATO 1 μM for 4 h, to downgrade PML::RARα [33], observing an increase in glycolysis, that was reversed by the addition of 1μM AKT inhibitor (Fig. 2B). Similar results, indicating the inhibition of glycolysis by AKT in APL cells, were obtained using NB4 cells, a cell line derived from a PML::RARα positive APL patient (Fig. 2C), and in PR9 cells induced to express PML::RARα by ZnSO_4_ treatment (Supplementary Fig.-S2A).

By western blot analysis we measured the levels of AKT and its phosphorylation at threonine in position 308 (Thr-308), a marker of AKT protein kinase activation, in primary APL blasts and in PML::RARα + NB4 and PR9 cell lines treated with ATO or with ATO + AKT inhibitor. We found that AKT protein is almost constant following PML::RARα degradation induced by ATO treatment. However, the degradation of PML::RARα by ATO, appears associated to AKT kinase activation, as shown by the increase of the phosphorylation at Thr-308, which is abrogated by the combination treatment of ATO with AKT inhibitor (Fig. 2D and Supplementary Fig.-S2B). Overall, these results suggest that PML::RARα oncoprotein inhibits glycolysis, whereas treatment with ATO, by inducing PML::RARα degradation and AKT kinase function, restores it. We observed a statistically significant increase of basal glycolysis in ATO treated PML::RARα+ cells, that is inhibited by treatment with ATO + AKT inhibitor. In contrast, in MT control cells ATO treatment slightly subsided glycolysis, which was further decreased by AKT inhibition (Supplementary Fig.-S2A and S2B). To note that, in the absence of PML-RARa, in MT cells, ATO induces a slight diminution of glycolysis, demonstrating that ATO per se does not induce glycolysis. Altogether, our data indicates that AKT degradation by PML::RARα is mechanistically responsible for glycolysis inhibition in APL blasts.

### PML::RARα expressing cells show a strong dependency on long chain fatty acids consumption

**Fig. 3:**
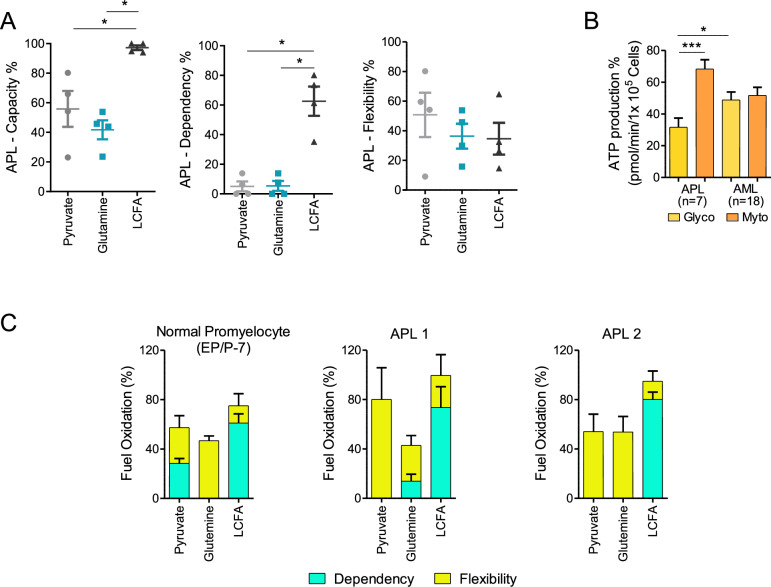
Evaluation of the mitochondrial fuel source. **A** Evaluation of the mitochondrial fuel used by four primary fresh APL blasts using the XF Myto Fuel Flex Test. The graphs show dependency, capacity, and flexibility of the cells to oxidize three mitochondrial fuels: pyruvate, glutamine and long-chain fatty acids (LCFAs). Statistical analysis was performed using the non-parametric Mann Whitney t test, * *p* ≤ 0.05. **B** Percentage of Glycolytic and mitochondrial ATP production in APL and AML patient’s samples measured using the ATP rate assay. Statistical analysis was performed using the Student’s t-test. * *p* ≤ 0.05, *** *p* ≤ 0.0005. **C** Evaluation of the mitochondrial fuel used by normal promyelocytes (hematopoietic precursors /progenitors at day 7, EP/P-7) derived from a pool of cord blood cells differentiated into granulocytes; and primary APL blasts isolated from two patients with the XF Myto Fuel Flex Test (N° 4 and 5 from Supplementary Table S1).

As shown in Fig. 1C-rigth and Supplementary Table-S3, PML::RARα expression in PR9 cells increases mitochondrial respiration. When compared to MT control cells, ZnSO4-induced PR9 cells (PML::RARα + ) show a strong dependency on long chain fatty acids (LCFA) consumption, as indicated by the exclusivity of a larger acute response under conditions of high LCFA demand (ETO), and not pyruvate (UK5099) and glutamine (BPTS) (Supplementary Fig. S3A). Primary APL blasts retain a high capacity to use LCFA and depend mainly on them for mitochondrial respiration (LCFA: 63 ± 20% *vs.* glucose: 5 ± 7% and glutamine: 5 ± 7%) (*p* < 0.0001) (Fig. 3A). When challenged, APL blasts display the ability to switch to pyruvate or glutamine to meet their energetic needs when the other pathways are inhibited (LCFA: 35 ± 21% *vs.* pyruvate: 51 ± 30% and glutamine: 36 ± 17%) (Fig. 3A). Of note, as mentioned before, glycolysis enzymes are present and, once produced, pyruvate is efficiently utilized. Thus, AKT inhibition by PML::RARα, with inefficient primary cytoplasmic glycolysis and subsequent pyruvate scarcity must be the cause of the inability to utilize the glycolytic pathway for APL’s cells metabolic needs. In primary blasts from seven APL patients’, 68% ± 14 of the ATP was produced by OXPHOS and 32% ± 14 by glycolysis (*p* = 0.0008) (Fig. 3B), whereas in eighteen primary blasts from AML patients’ 49% ± 22 of the ATP was produced by OXPHOS and 49% ± 22 by glycolysis (AML *vs.* APL glycolysis *p* = 0.02) (Fig. 3B).

These results indicate that the APL patients’ blasts rely mainly on LCFA oxidation with loss of the glycolytic dependence shown by normal promyelocytes (EP/P-7) (Fig. 3C) and differently from other AMLs subtypes.

### PML::RARα promotes TCA

**Fig. 4:**
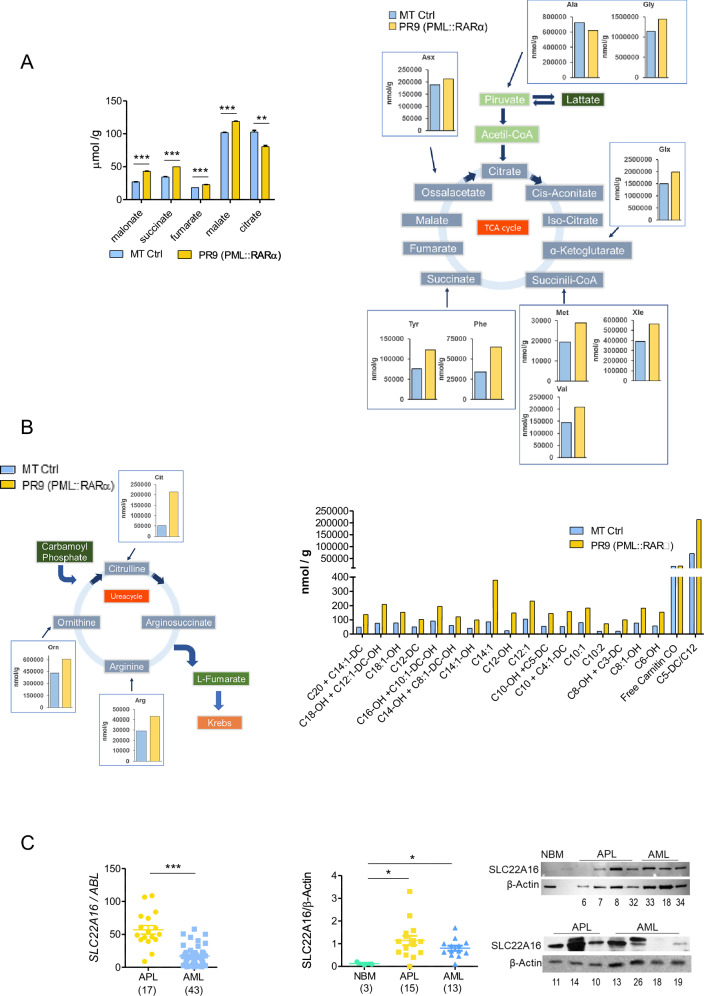
PML/RARα promotes tricarboxylic cycle acid (TCA) activity. **A, left** The histogram shows the levels of TCA cycle intermediates in MT (control) and PR9 (PML::RARα^+^) cells cells from two independent biological replicates, as determined by mass spectrometry. **A, right** Schematic representation of the TCA cycle and the entry points of various amino acids, whose amount increases in PML::RARα^+^ cells fostering the TCA cycle. Data represents one biological replicate. Statistical significance was assessed using Student’s t-test: **p ≤ 0.005, ***p ≤ 0.0005. **B, left** Schematic representation of the metabolic interplay between the urea cycle and the aspartate–argininosuccinate shunt of the TCA cycle. The histogram shows the intracellular concentrations of L-Citrulline (Cit), L-Arginine (Arg) e L-Ornithine (Orn) amino acids in MT (control) and PR9 (PML::RARα^+^) cells, measured by mass spectrometry from one biological replicate. **B, right** Intracellular acylcarnitine profiles in MT (control) and PR9 (PML::RARα^+^) cells from one biological replicate. Profiles include free carnitine (C0), short-chain acylcarnitines (C3–C6), and medium- to long-chain acylcarnitines (C8–C22). Values are expressed as nmol/g and normalized to total protein content. Metabolite levels were quantified by LC-MS/MS. **C, left** mRNA expression levels of SLC22A16 (also known as CT2) in primary APL and AML blasts, as well as in normal bone marrow (NBM) cells. **C, right** Protein expression levels of SLC22A16 (CT2), analyzed by Western blot in the same samples. Statistical analysis was performed using the non-parametric Mann Whitney *t* test, * *p* ≤ 0.05. Patient identifiers are listed at the bottom of the blot and correspond to those in Supplementary Table S1. Protein expression was quantified by densitometric analysis and normalized to β-actin or GAPDH. Statistical analysis was performed using the non-parametric Mann Whitney t test, * *p* ≤ 0.05; ***p < 0.0005.

These data let us hypothesize a major involvement of the Krebs’ cycle or tricarboxylic acid cycle (TCA) in APL cell’s metabolism. Thus, we studied, using mass spectrometry, the levels of the intermediates of the TCA, acylcarnitines, intermediates of Urea cycle and other amino acids (AA). In comparison to MT control cells, PR9 cells (PML::RARα + ) showed a significantly increase in the levels of succinate (50 ± 0.04 *vs.* 34 ± 0.1, *p* = 0.003), fumarate (23 ± 0.1 *vs.* 18 ± 0.1, *p* = 0.0004), malate (102 ± 0.6 *vs.* 119 ± 0.5, *p* = 0.001) and a slight decrease in the citrate concentration (103 ± 3 *vs.* 80 ± 3, *p* = 0.02), indicating an activation of the Krebs cycle (Fig. 4A). The increased TCA activity in PML::RARα+ cells, is in line with the high LCFA oxidation levels and the increase in OXPHOS observed in APL blasts and PML::RARα+ cells (Fig. 3A, Fig. 1C-right and Supplementary Fig.-S3A). Of note, in PML::RARα+ cells citrate quantity is conserved, and glycolysis is downgraded. That sets apart APL’s metabolic background, since in cancer cells in general, citrates at a low concentration allow glycolysis even in the presence of oxygen (Warburg effect) [34].

We also measured increased concentration of malonate (PR9: 43 ± 0.4 *vs.* MT: 27 ± 0.1, *p* = 0.0004) (Fig. 4A left), a known inhibitor of the succinate dehydrogenase, inhibiting conversion of succinate in fumarate. What appears to be an impediment to the Krebs cycle from proceeding is eluded by the intensification of AA uptake and increased intracellular concentration of several AA (Fig. 4A right and Supplementary Fig. S3B) and by the activation of the Urea cycle, indicated by the increase in the concentrations of citrulline (PR9:51123 *vs.* MT:213128), arginine (PR9:29091 *vs.* MT:43182) and ornithine (PR9:431700 *vs.* MT:605912). That provides fumarate to the Krebs cycle by the aspartate-argininosuccinate shunt (Fig. 4B left). The slightly reduced concentrations of citrate (PR9: 80 ± 3 *vs.* MT:103 ± 3, *p* = 0.02) that, as a consequence, would reduce fatty acids synthesis fit in a context where the glycolysis is inhibited, and LCFA consumption seems to be of primary importance for ATP production. That is in line with the increased concentration of the acylcarnitines when compared to MT control cells (Fig. 4B right).

### PML::RARα expression favors LCFA catabolism in APL cells

To better characterize the mechanics of the metabolic adaptation driven by PML::RARα, we analyzed the mRNA and protein levels of key metabolic enzymes in primary blasts from APL and AML patients and in PR9/MT cells treated with ZnSO_4_ for 6 h. Pyruvate dehydrogenase (PDHA1), involved in the synthesis of acetyl-CoA derived from glycolysis, to be used in TCA, is unchanged in these cells: PDHA1 mRNA (APL: 0.7 ± 0.5, *n* = 11; AML 0.9 ± 0.4, *n* = 43, *p* = 0.04) (Supplementary Fig. S4A); PDHA1 protein (APL: 0.6 ± 0.4, *n* = 11; AML 0.7 ± 0.3, *n* = 43, *p* = 0.4) (Supplementary Fig. S4B); PDHA1 protein (PR9 1.2 ± 0.2; MT cells 1.2 ± 0.7, *p* = 0.9) (Supplementary Fig. S4C).

The mRNA and protein expression levels of the carnitine transporter SLC22A16, also known as CT2, necessary for the transport of carnitine into the cytosol, are significantly higher in APL blasts: SLC22A16 mRNA (APL: 57 ± 27, *n* = 17; AML 17 ± 15, *n* = 43, *p* = 0.0001) (Fig. 4C left); SLC22A16 protein (APL: 1.2 ± 0.48), *n* = 15; AML: 0.7 ± 0.3, *n* = 13, *p* = 0.04; NBM 0.1 ± 0.03, *n* = 3, *p* = 0.03 (Fig. 4C right). The expression of PML::RARα in PR9 cells significantly induces an increase in SLC22A16 mRNA, if compared to MT control cells (Supplementary Fig. S4D). As a whole, these data suggest that the main supplier of acetyl-CoA to the tricarboxylic acid cycle is not glycolysis but the oxidation of LCFA, underlining its role as a possible therapeutic target in APL.

### Combination treatment with venetoclax and azacitidine (VTX + AZA) efficiently targets PML::RARα positive cells

**Fig. 5:**
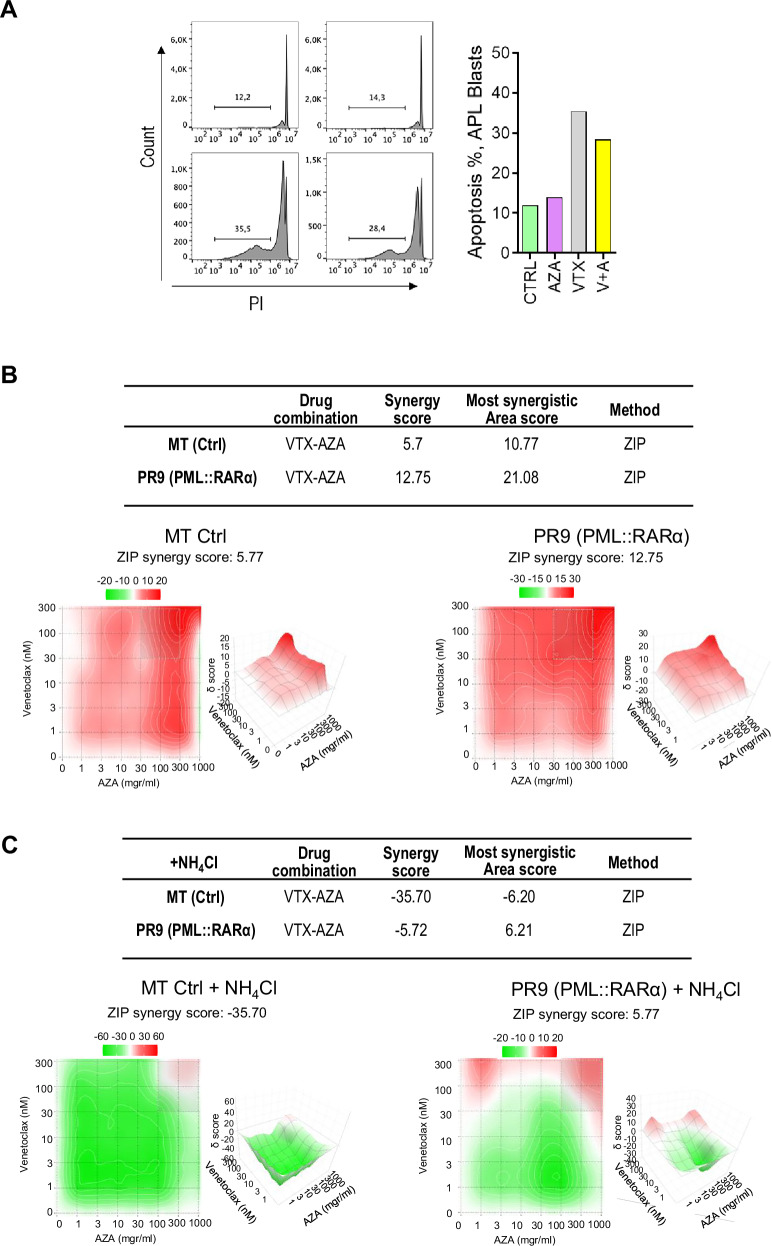
Synergistic activity of venetoclax and azacytidin on PML::RARA+ cells. **A** Apoptosis was assessed by flow cytometry using propidium iodide (PI) staining to measure sub-G1 DNA content, which reflects the population of cells undergoing DNA fragmentation—a hallmark of late apoptosis. This analysis was performed on blast cells from one APL patient, either untreated or treated with Azacitidine (AZA) and Venetoclax (VTX) for 48 h. **B** Two dimension (2D) and 3D synergy map for the combination of VTX (0 to 300 nM) and AZA (0 to 1000 ng/ml) analyzing cell grow by MTT assay. Data represent two independent biological replicates performed in MT and PR9 (PML::RARα^+^) cells. The ZIP score (∂-score) for each drug combination is indicated by the color code given above the panel grid (synergistic and antagonistic dose regions in red and green colors, respectively). **C** Two dimension (2D) and 3D synergy map for the combination of VTX (0 to 300 nM) and AZA (0 to 1000 ng/ml) plus NH_4_Cl, analyzing cell grow by MTT assay. Data represent two independent biological replicates performed in MT and PR9 (PML::RARα^+^) cells. The ZIP score (∂-score) for each drug combination is indicated by the color code given above the panel grid (synergistic and antagonistic dose regions in red and green colors, respectively). ZIP score >10 indicates synergism; ZIP score between -10 and 10 indicates additivity; and ZIP score <-10 indicates antagonism. The panels were obtained by Synergy Finder analysis (https://synergyfinder.fimm.fi/synergy/synfin_docs/).

Overexpression of anti-apoptotic proteins BCL2, BCL-xL, MCL-1 causes resistance to chemotherapy in AML cells [35, 36]. We measured their expressions in MT and PR9 cells, observing a significant increase in the presence of PML::RARα (Supplementary Fig. S4E). The reliance on OXPHOS and the low glycolytic capacity of PML::RARα+ cells suggest sensitivity to BCL-2 inhibitors. VTX (a selective BCL-2 inhibitor) plus AZA (a hypomethylating agent that also suppresses anti-apoptotic proteins like MCL-1) [37, 38], has become the standard of care for newly diagnosed AML patients, unfitted for intensive chemotherapy [39]. We treated primary blasts from an APL patient with VTX, AZA, or a combination of both, demonstrating the sensitivity of APL cells to this therapeutic approach (Fig. 5A). This combination may also benefit APL patients resistant to ATO and ATRA. To test this hypothesis, we treated MT control cells and PML::RARα + , PR9 cells with VTX + AZA and assessed cell growth via MTT assay. Synergism scores were 12.7 (max 21.47) in PR9 cells and 5.7 (max 10.77) in MT cells (Fig. 5B), indicating a stronger synergistic effect in PML::RARα+ cells. Notably, cleaved caspase-3 and PARP were detectable after 48 h of VTX or AZA treatment in PR9 cells but absent in MT controls, with PARP cleavage further increasing upon VTX + AZA combination (Supplementary Fig. S5A). These results confirm terminal apoptosis activation and APL cell sensitivity to VTX + AZA.

To explore the apoptotic mechanism, we analyzed MCL-1 and BCL-xL expression. In MT cells, VTX + AZA treatment increased both proteins, while in PML::RARα+ cells their levels markedly decreased (Supplementary Fig. S5B). JC-1 staining of mitochondrial membrane potential (ΔΨm) by flow cytometry showed AZA had a stronger impact than VTX in inducing apoptosis, while their combination exerted a pronounced synergistic effect in PR9 cells compared to MT controls (Supplementary Fig. S5C). These findings confirm that PML::RARα enhances mitochondrial depolarization upon treatment, sensitizing cells to apoptosis.

Since autophagy can synergize with apoptosis under metabolic stress [40], we evaluated the consequences of the autophagy inhibition in the synergistic response of PML::RARα_+_ cells to VTX + AZA treatment. MT control cells and PML::RARα + PR9 cells were treated with VTX + AZA in the presence of NH_4_Cl (an inhibitor of autophagy) and assessed for cell growth via MTT assay. Notably, pharmacological inhibition of autophagy with NH_4_Cl reversed the synergistic effect of VTX + AZA in both cell types, PR9 cells -5.72 (max 6.21) and MT cells -35.7 (max -6.20) converting it into a strong antagonism (Fig. 5C). These findings suggest a role of autophagy in modulating the sensitivity of APL cells to the VTX + AZA combination.

### LCFA catabolism and glycolysis are enhanced in NB4 cells resistant to ATO

We individuated metabolic changes and possible vulnerabilities in two subclones of NB4 cells resistant to ATO (NB4-ATOR), compared to relative control clones [41].

**Fig. 6:**
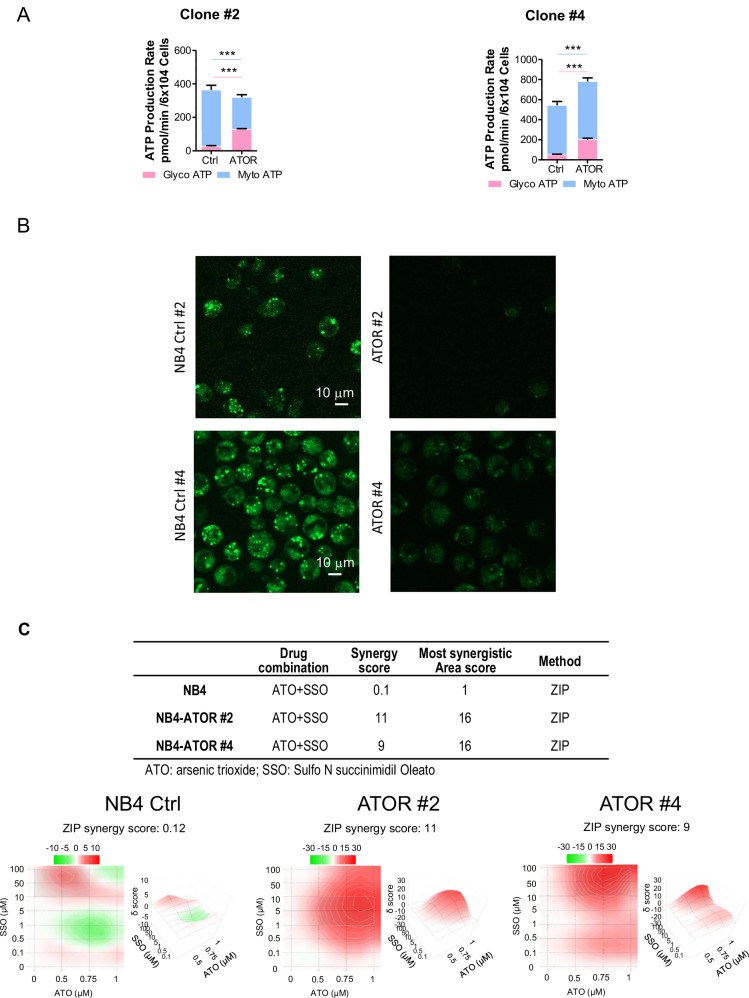
Metabolic characterization of ATO-resistant NB4 cells (ATOR #2 and #4 clones) and control cells. **A** glycolytic and mitochondrial ATP production rate using the XF Real-Time ATP Rate Assay. Data represent two independent biological replicates performed in MT and PR9 (PML::RARα^+^) cells. **B** BODIPY staining. Representative images (20x magnification) of (up) NB4 Ctrl #2 *vs.* ATOR #2 and (down) in NB4 Ctrl #4 *vs.* ATOR #4, stained with BODIPY 493/503 (D-3922). Scale bar 10 μm. **C** Two-dimension (2D) and (3D) synergy map for the combination of SSO (CD36 inhibitor) (0 to 100 µM) and ATO (0 to 1 µM) analyzing cell growth by MTT assay. Data represent two independent biological replicates performed in MT and PR9 (PML::RARα^+^) cells. The ZIP score (∂-score) for each drug combination is indicated by the color code given above the panel grid (synergistic and antagonistic dose regions in red and green colors, respectively). ZIP score >10 indicates synergism; ZIP score between -10 and 10 indicates additivity; and ZIP score <-10 indicates antagonism. Panels **A** and **B** and **C** were obtained by Synergy Finder analysis (https://synergyfinder.fimm.fi/synergy/synfin_docs/).

ATO resistant, (ATOR) Clone #2 exhibited inferior basal OXPHOS levels and ATP production than control cells (Ctrl #2) (Supplementary Fig. S6A). On the contrary, ATOR Clone #4 showed higher OXPHOS levels and ATP production. ECAR measurements underlined an increase in basal glycolysis in both the ATOR clones (Supplementary Fig. S6B). However, the glycolytic capacity was unchanged in ATOR #2, whereas it was increased in ATOR #4 cells. In contrast, the glycolytic reserve was lower in ATOR #2 and higher in ATOR #4. Taking together these data suggest a reactivation of the glycolytic pathway in ATO resistant APL clones, further supporting the increased production of glycolytic ATP in these cells (Fig. 6A). In addition, the ATP-Glo cell viability assays showed a greater dependence of both ATOR clones to diminished concentrations of glucose (Supplementary Fig. S6C).

The ATOR clones #2 and #4 cell viability was also extremely sensitive to the inhibition of mitochondrial β-oxidation, by treatment with the mitochondrial carnitine palmitoyltransferase-1 (CPT1) inhibitor perhexiline (Supplementary Fig. S7A). A higher LCFA consumption compared to control cells was revealed by Bodipy staining (Fig. 6B), a finding that paralleled a significant increase of the SLC22A16 protein in ATOR #4 clone (Supplementary Fig. S7B). Our data suggest that these two ATO resistant NB4 clones rely to a higher extent to the β-oxidation of LCFA than in control sensitive NB4 cells. That is in line with the increased concentrations of the acylcarnitines in ATOR clones #2 and #4 when compared to NB4 control cells (Supplementary Fig. S7C).

Since our findings suggest that ATOR clones exhibit a high dependence on fatty acid metabolism, we downregulated fatty acid uptake using the CD36 inhibitor. In ATOR cells the synergism score of the association ATO + SSO was 9 (#Cl2) and 11 (#Cl4) with a maximum of 16 versus 0.1 with a maximum of 1 in control sensitive NB4 cells (Fig. 6C). These results confirm the greater dependence of the ATOR clones on fatty acids and underscore the potential of CD36 inhibition as a therapeutic strategy to overcome ATO resistance.

Of note that VTX + AZA treatment was also effective in both ATOR #2 and ATOR #4 clones (Supplementary Fig. S8).

## Discussion

We previously reported that the oncogenic protein AKT, responsible driver of the glycolytic up regulation in cancer cells [42, 43], is downregulated in APL cells. PML::RARα, via HSP90 promoter inhibition, induces degradation of AKT by phosphorylation at p473-AKT and ubiquitination [32]. In the present study, characterizing APL cells metabolic landscape, we demonstrate that the expression of PML::RARα, by favoring the degradation of AKT, is responsible for the reduced reliance on glycolysis. PML::RARα expression is also responsible for the increase of the mitochondrial respiration powered by other fuels, in particular LCFA. To note that glycolysis enzymes are present in APL blasts and, once produced, pyruvate is efficiently utilized in those cells. Thus, APL cells inefficiency in utilizing the glycolytic pathway for their metabolic needs depends solely on AKT inhibition and the consequent pyruvate scarcity. Our data indicate that the APL patients’ blasts rely mainly on LCFA oxidation, with loss of the glycolytic dependence observed in normal promyelocytes. Patients’ samples from other AML subtypes show a great variability in glycolytic values [28], but APL cells consistently feature a profound inhibition of glycolysis (Fig. 1A). Whereas normal HSCs depend fundamentally on glycolysis and have the capacity to increase it in stress conditions [44–46], APL cells cannot resort to glycolysis as alternative energy source. We demonstrate that, in APL, the main supplier of acetyl-CoA to TCA is the oxidation of LCFA, revealing its role as a possible therapeutic target. Importantly, the lower glycolytic capacity observed in APL cells indicates a therapeutic window for targeting selectively these cells with OXPHOS inhibitor drugs as venetoclax® [47] (Fig. 5), living normal HSCs unscathed.

A considerable amount of research has shown the importance of AA in cancer metabolism. In addition to representing an alternative fuel, AA are involved in the redox balance, biosynthetic support, and homeostatic maintenance [48, 49].

Glutamine and branched-chain amino acids (BCAA; valine, leucine and isoleucine) can easily fuel the TCA cycle (anaplerosis) and fatty acids synthesis [49]; glycine, glutamine, aspartate, serine and methionine, by different pathways, serve as carbon and nitrogen donors for purine synthesis [50, 51]; glutamine, glutamate, methionine, and phenylalanine are relevant for non-essential amino acids (NEAA) synthesis [52]. In addition, amino acids produce metabolites able to foster tumor onset and progression [53].

Proliferation and metabolism of cancer cells determine the accumulation of reactive oxygen species (ROS), which is also balanced by the production of NADPH through the folate cycle and in particular by serine-driven one-carbon metabolism [54]. We previously demonstrate that PML::RARα disrupts NRF2 function, sensitizing cells to oxidative stress [8].

In PML/RARa+ cells, we observed increased AA uptake and intracellular accumulation of citrulline, arginine, and ornithine, suggesting Urea cycle activation, which may fuel the TCA cycle. Notably, VTX inhibits AA consumption, promoting ROS accumulation [46]. We ascertained that PML::RARα induces BCL-2, MCL-1 and BCL-xL proteins (Supplementary Fig. S4E). Building on our findings and prior research [47] we challenged APL cells with VTX (BCL-2 inhibitor) and AZA (MCL-1/BCL-xL inhibitor), which also disrupts mitochondrial metabolism and enhances oxidative stress [37].

Synergy scores revealed an excess response due to drug interaction, suggesting a mutation-driven mechanism. Overall, PML::RARα+ cells showed high sensitivity to this treatment, with reduced MCL-1 and BCL-xL expression in the presence of VTX + AZA. Their combination overwhelms antioxidant defenses, leading to mitochondrial damage and apoptosis.

These findings underscore the potential of this combination as a targeted therapy for relapsed or resistant APL. It is well known the association of fats and AML, in terms of epidemiology and clinic, since high body mass index (BMI) predispose to the disease and is associated with a more severe prognosis [9, 55]. Moreover, bone marrow adipocytes can support the survival and proliferation of AML blasts [56]. In the leukemic bone marrow microenvironment, AML blasts release cytokines able to stimulate hormone-sensitive lipases inducing lipolysis and FA release that can feed leukemic cells [57]. The increased risk of developing APL in subjects with high BMI is confirmed by a wide range of studies across different countries and populations with significantly different dietary regimens and prevalence of obesity [58]; an elevated BMI is associated also with a poor prognosis in APL [59]. As expected, the persistence of *PML::RAR*α in ATO resistant clones relates to the great variability in their metabolic landscape and behavior when challenged with specific inhibitors. But over the differences, our data suggest that both ATO resistant clones: 1- rely to a higher extent to the β-oxidation of LCFA and 2- reactivate glycolysis as compared to control ATO sensitive NB4 cells and PR9 PML::RARα positive cells. We previously demonstrated that MCL-1 regulates glycolysis via direct interaction with Hexokinase2 [28], indicating MCL-1 inhibition by AZA as a possible mean to overcome resistance. Downregulating fatty acid uptake using CD36 inhibitor SSO inhibits growth of ATO resistant clones. These data suggest that targeting the fatty acid uptake may be a useful therapeutic strategy for resistant and relapsed APLs.

By characterizing for the first time the APL metabolic background, we demonstrate that PML::RARα inhibits glycolysis via AKT degradation, promotes TCA, and favors LCFA catabolism, exposing APL cells to strong dependency and vulnerabilities to OXPHOS inhibition. Selective dependence on OXPHOS, associated to low glycolytic capacity indicates BCL-2 inhibitors (VTX) plus AZA, the standard of care for patients with newly diagnosed AML unfitted for intensive chemotherapy [39], as a therapy for resistant and relapsed APL patients. Our hypothesis is corroborated by previous studies reporting the in vitro sensitivity of APL blasts to BCL-2 inhibitors and the therapeutical efficacy of VTX in relapsed/resistant APL patients [47, 60]. We also directly characterized two NB4-ATO resistant cell lines and found enhanced LCFA catabolism and reactivation of glycolysis. In those cells VTX + AZA is a highly efficient therapy, and targeting CD36 function inhibits cells growth, indicating a possible role in association.

VTX + AZA treatment may act through autophagy as well as apoptosis: pharmacological inhibition of autophagy with NH_4_Cl reversed the synergistic effect of VTX + AZA in PML:: RARα+ and control cells, converting it into strong antagonism, particularly in control cells. However, the less severe antagonism observed in PML:: RARα+ cells under these conditions implies that additional pro-death pathways may be engaged downstream of PML:: RARα + , partially compensating for the loss of autophagy. These results collectively suggest that functional autophagy is essential for the full cytotoxic synergy of VTX and AZA, and that PML:: RARα may prime cells for enhanced death through both autophagy-dependent and -independent mechanisms.

## Supplementary information

Supplementary Material

Supplementary Tables

## Data Availability

The datasets generated during and/or analyzed during the current study are available from the corresponding author on reasonable request.
