# Supplementary material for: PML::RARα+ myeloid cells display metabolic alterations that can be targeted to treat resistant/relapse acute promyelocytic leukemias
Source: Leukemia. 2025 Sep 10;39(11):2708–20. doi: 10.1038/s41375-025-02738-9 (PMC12589136; doi:10.1038/s41375-025-02738-9)
Supplement: Supplementary file 1 — Supplementary Material [file 41375_2025_2738_MOESM1_ESM.pdf]

Supplementary Figures

Supplementary Figure S1. Expression of key glycolytic enzymes in PML::RARα cells. (**A**) HK2, PKM2 and PFKP protein expression were analyzed in MT (control) and PR9 cells (a zinc-inducible PML::RARα model constructed from the U937 cell line) treated with ZnSO4 100 μM for 24 hs. Data represent one biological replicates performed in MT and PR9 (PML::RARα^{+}) cells. Protein values were obtained by densitometry and reported in the histogram after normalization with β-actin.

Supplementary Figure S2 PML::RARα inhibits glycolysis via AKT degradation. (**A**) MT (Ctrl) and PR9 (PML::RARα+) cells were treated with 1 μM ATO for 4 hours and with 1 μM VIII inhibitor (AKT inhibitor) for the last 30 min. Histograms represent basal glycolysis and compensatory glycolysis obtained with the glycolytic rate assay. Data represent two independent biological replicates. (**B**) PML::RARα, AKT and p-AKT308 protein levels in MT (Ctrl) and PR9 (PML::RARα+) cells. Protein values were obtained by densitometry and reported in the histogram after normalization with β-actin. Statistical analysis by Student's t-test. * $p \leq 0.05$, ** $p \leq 0.005$, *** $p \leq 0.0005$.

Supplementary Figure S3. Evaluation of the mitochondrial fuel source. (**A**) Evaluation of the mitochondrial fuel source used by MT (Ctrl) and PR9 (PML::RARα+) cells using the Substrate Oxidation Stress Test. Histogram represent the acute response for substrate demand after sequential compound injections of specific pyruvate, glutamine and LCFA inhibitors (UK5099, BPTES or Etomoxir, respectively). Data represent two independent biological replicates. (**B**) The histograms represent the Amino acids concentration measured from MT (Ctrl) and PR9 (PML::RARα+) cells by mass spectrometry. Data represent one biological replicate.

Supplementary Figure S4 Proteins involved in metabolic and apoptotic regulation. (**A**) mRNA and (**B**) protein expression levels of pyruvate dehydrogenase (PDH) in primary blasts obtained from the indicated number of APL and AML patients. The numbers at the bottom of the western blot identify the patients in table N° 1. Protein values were analyzed by densitometry scanning and reported in the histogram after normalization with β-actin. (**C**) (**left**) *PDHA1* and (**right**) *SLC22A16* mRNA expression levels in MT (Ctrl) and PR9 (PML::RARα+) cells treated for 3 and 6 hours with ZnSO4. Data represent three independent biological replicates (**D**) BCL-2, MCL-1 and BCL-xL protein levels in MT (Ctrl) and PR9 (PML::RARα+) cells treated with ZnSO4 for the indicated times. Data represent three independent biological replicates. Protein values were obtained by densitometry and reported in the histogram after normalization with β-actin. Statistical analysis was performed using the Student's t-test. * $p \leq 0.05$.

Supplementary Figure S5 Synergistic activity of venetoclax and azacytidin on MT (Ctrl) and PR9 (PML::RARA+). (**A**) Western blots detecting cleaved caspase 3 and Parp 1. Data represent three independent biological experiments. (**B**) MCL-1 and BCL-xL protein levels in MT (ctrl) and PR9 (PML::RARA+) cells untreated and treated with Azacitidine (AZA) and Venetoclax (VTX) for 48 hours, C (Ctrl), A (AZA), V (VTX). Data represent three independent biological experiments (**C**) Apoptosis Analysis by JC-1, measuring mitochondrial membrane potential (ΔΨm) (Flow Cytometry) in MT (ctrl) and PR9 (PML::RARA+) cells untreated and treated with Azacitidine (AZA) and Venetoclax (VTX) for 48 hours. Data represent two independent biological experiments

Supplementary Figure S6. Metabolic characterization of ATO-resistant NB4 cells (ATOR #2 and #4 clones) and control cells. In the ATOR clone #2 (**Left**), clone #4 (**right**) and specific control cell lines we measured: (**A**) Mitochondrial respiration (OCR): histograms represent basal respiration (basal), maximal respiration (Max Resp), proton leak, ATP mitochondrial production and spare respiratory capacity (Spare Resp Cap). Data represent two independent biological experiments. Statistical analysis was performed using the Student's t-test. * $p \leq 0.05$, ** $p \leq 0.005$. (**B**) Glycolytic activity (ECAR). Histograms represent basal glycolysis, glycolytic capacity and glycolytic reserve by glycolytic rate assay. Data represent two independent biological experiments. (**C**) Cell viability, after 72 hrs, at normal concentration (2g/L), low concentration (0.1 g/L) or absence of glucose (0) by ATP glow assay. Data represent three independent biological experiments.

Supplementary Figure S7 ATO resistant NB4 clones (ATOR) are dependent on Fatty Acids (**A**) Cell viability measured using the CellTiter-Glo® Luminescent Cell Viability Assay in (left) NB4 Ctrl #2 *vs* ATOR #2 and (right) in NB4 Ctrl #4 *vs* ATOR #4. The cells were treated with 1,5 and 10 μM perhexiline for 72 hrs. Three independent experiments were performed. Data are presented as mean ± SD. **(B)** SLC22A16 protein expression in NB4 Ctrl #2 *vs* ATOR #2 and in NB4 Ctrl #4 *vs* ATOR #4 cells. Protein values were analyzed by densitometry scanning and reported in the histogram after normalization with β-actin. Data represent three independent biological replicates. Statistical analysis was performed using Student's t-test. * $p \leq 0.05$, ** $p \leq 0.005$. (**C**)**:** The histogram represent: - Intracellular acylcarnitines profiles obtained from (**Up**) NB4 Ctrl #2 *vs* ATOR #2 and (**Down**) NB4 Ctrl #4 *vs* ATOR #4 cells by mass spectrometry. Free carnitine (C0); Short-chain acylcarnitines (C3-C6); Medium- and long-chain acylcarnitines (C8-C22). Values are expressed as nmol/g. Data represent one biological experiment.

Supplementary Figure S8 Activity of venetoclax and azacytidin on NB4 Ctrl, ATOR#2 and ATOR#4 cells. Apoptosis Analysis by JC-1, measuring mitochondrial membrane potential (ΔΨm) (Flow Cytometry) in NB4 Ctrl, ATOR #2 and ATOR #4 cells, untreated and treated with Azacitidine (AZA) and Venetoclax (VTX) for 48 hours. Data represent two biological experiments.

Supplementary Figure S1

A

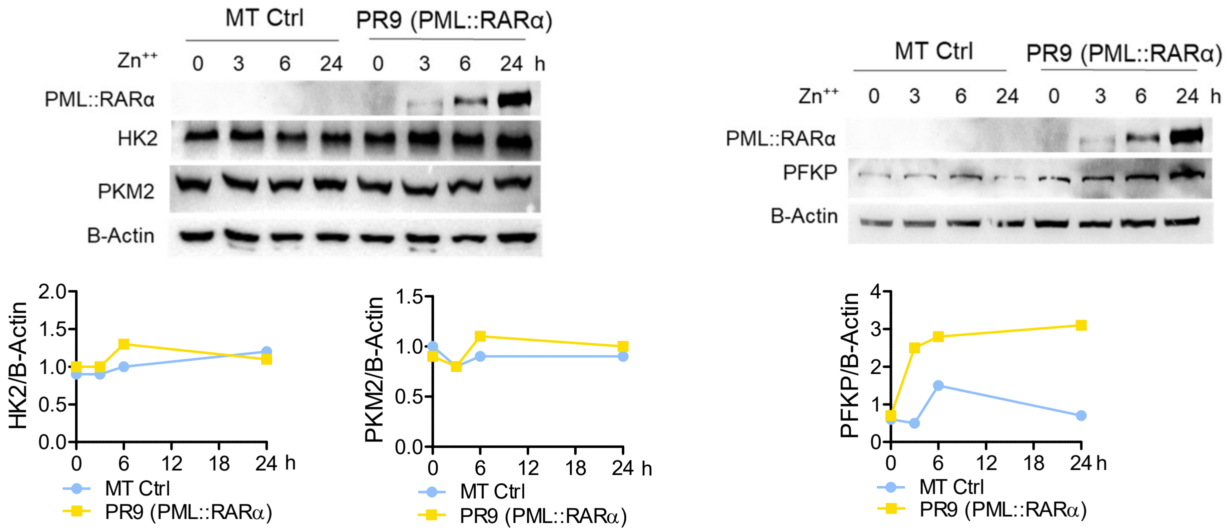

Supplementary Figure S2

A

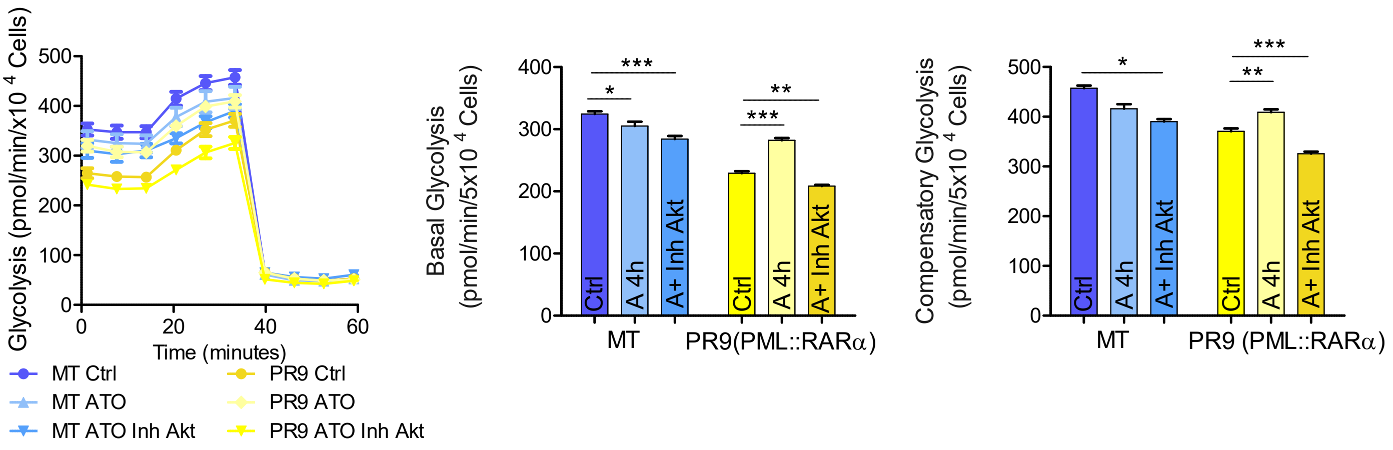

B

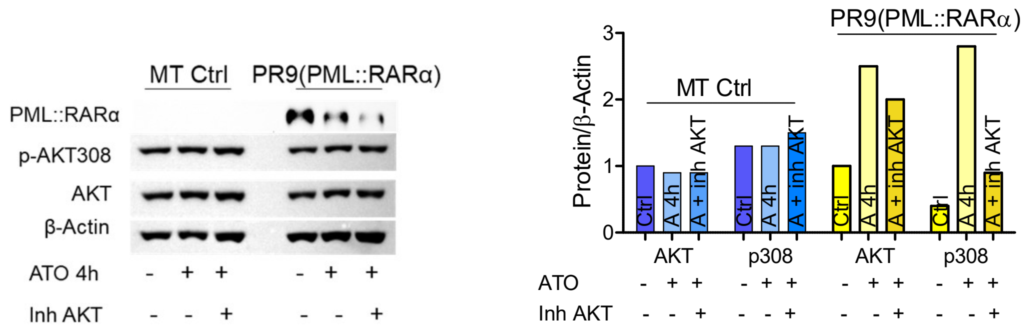

Supplementary Figure S3

A

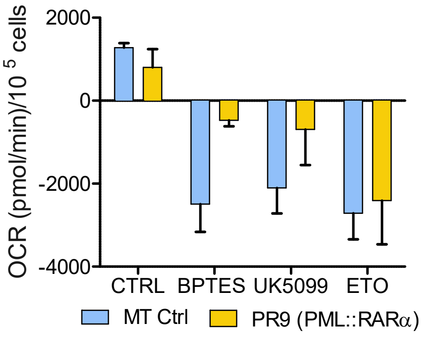

B

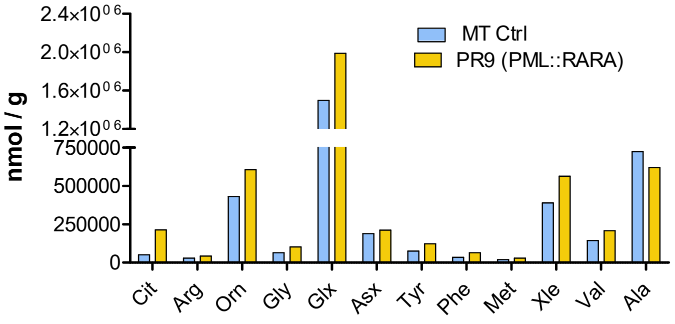

Supplementary Figure S4

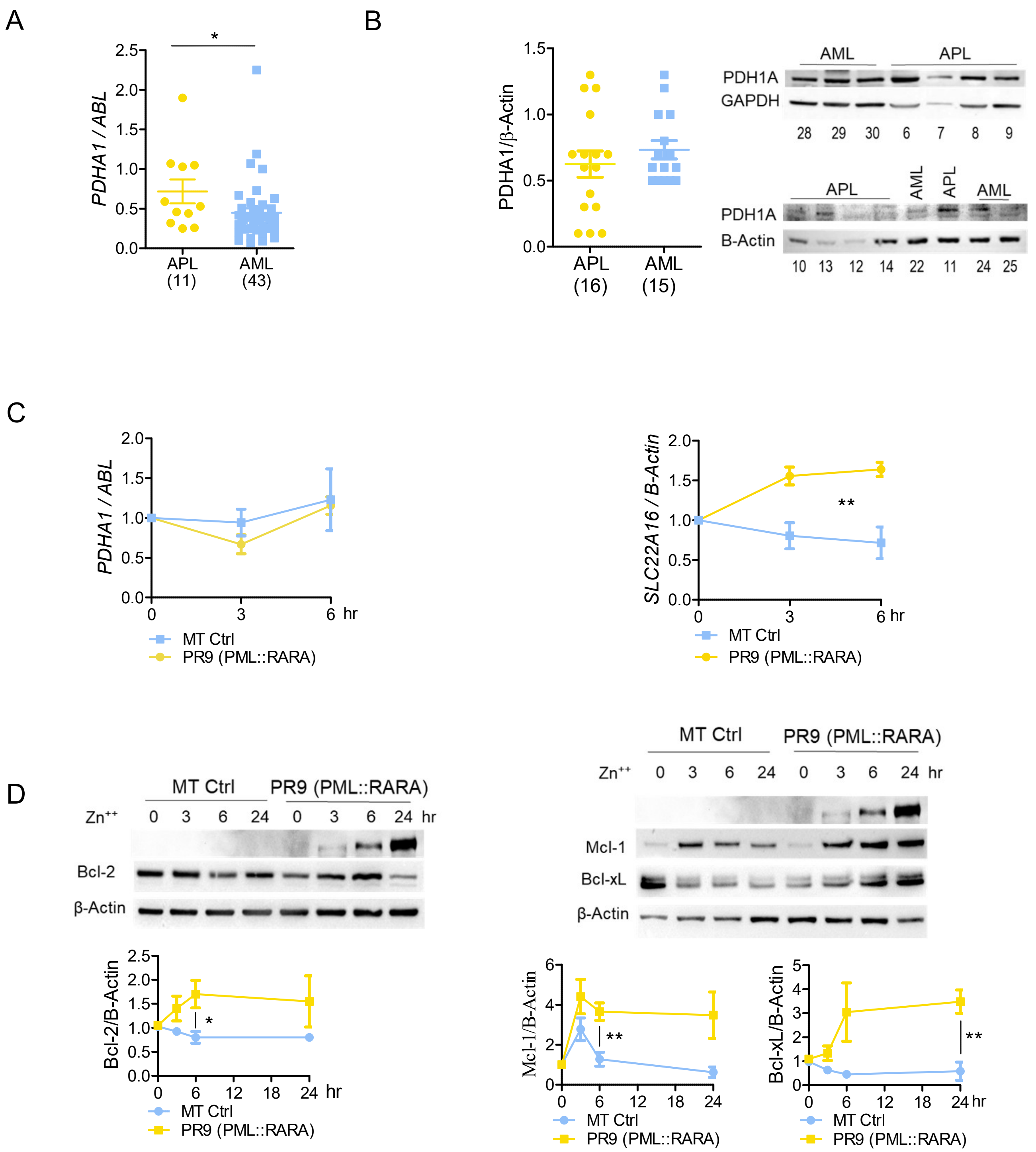

Supplementary Figure S5

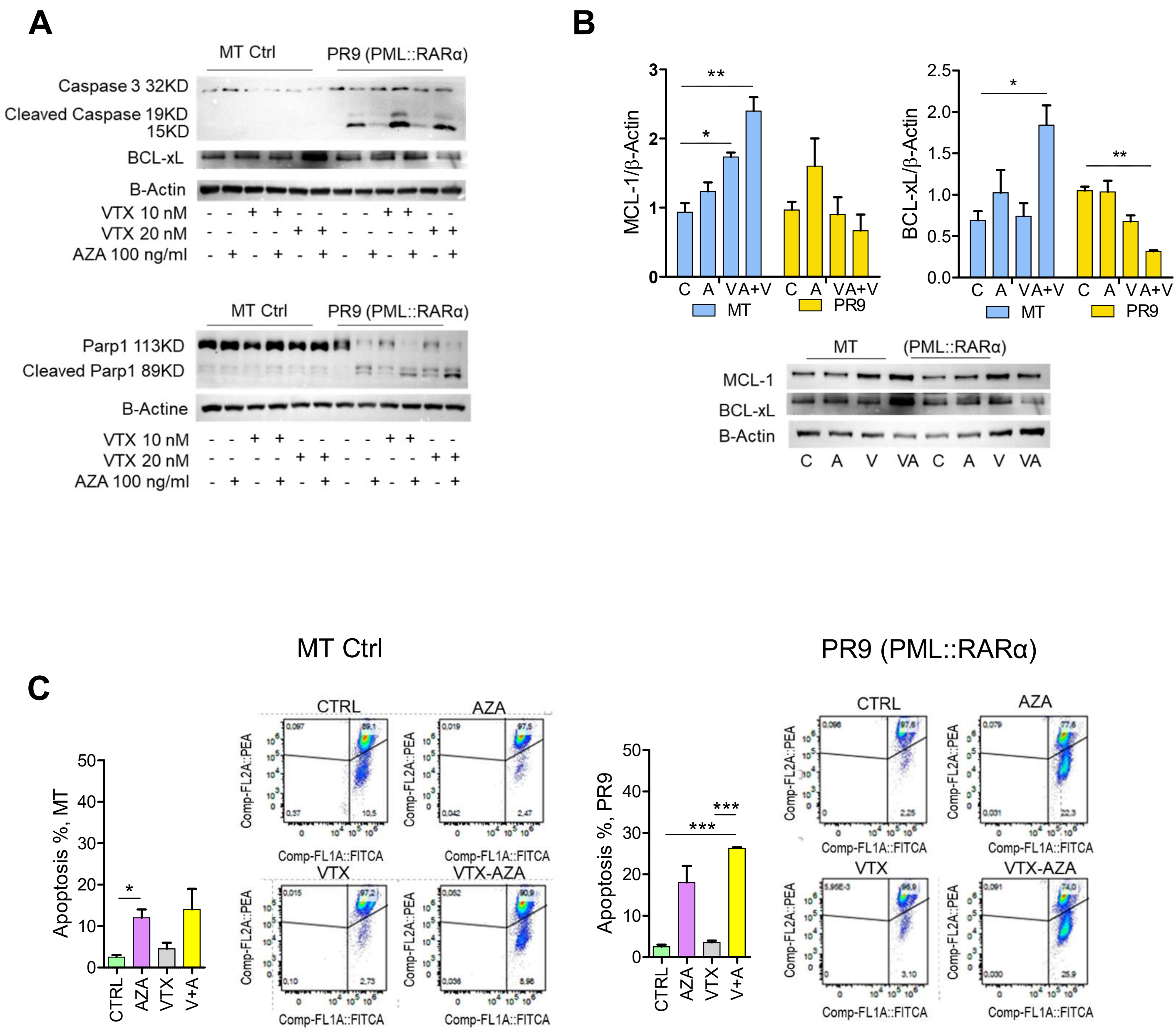

Supplementary Figure S6

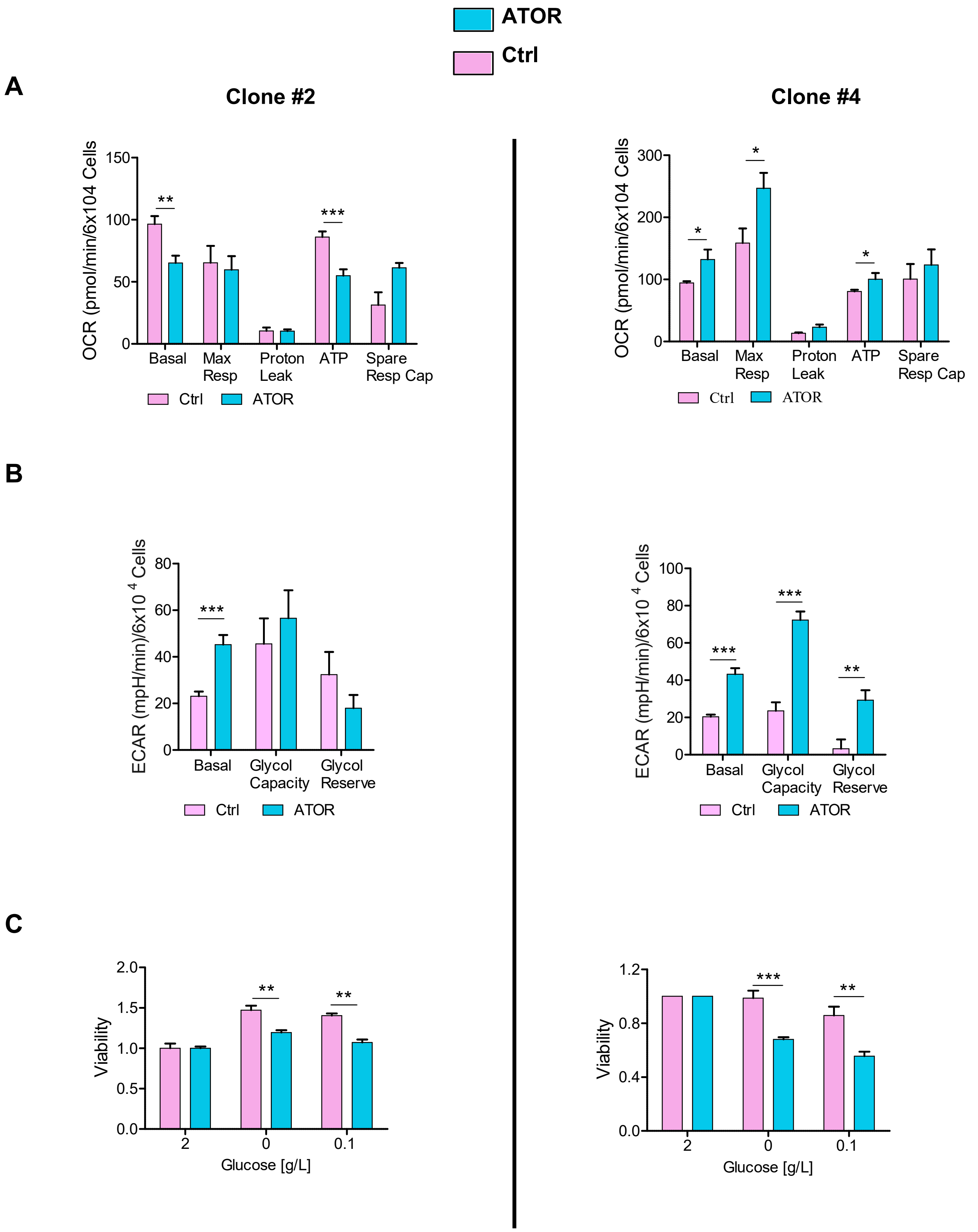

Supplementary Figure S7

A

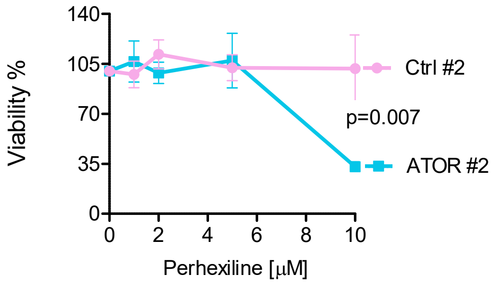

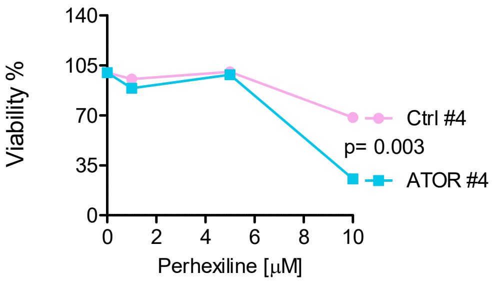

B

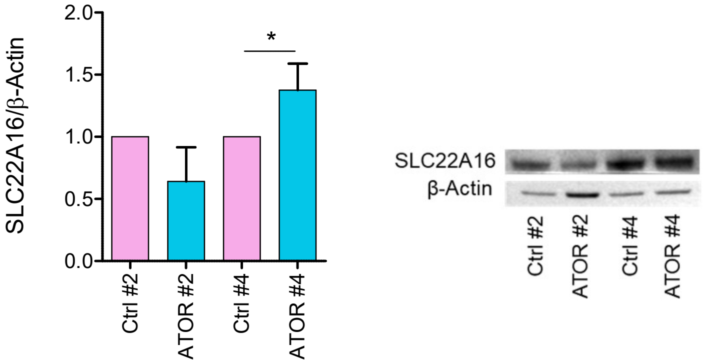

C

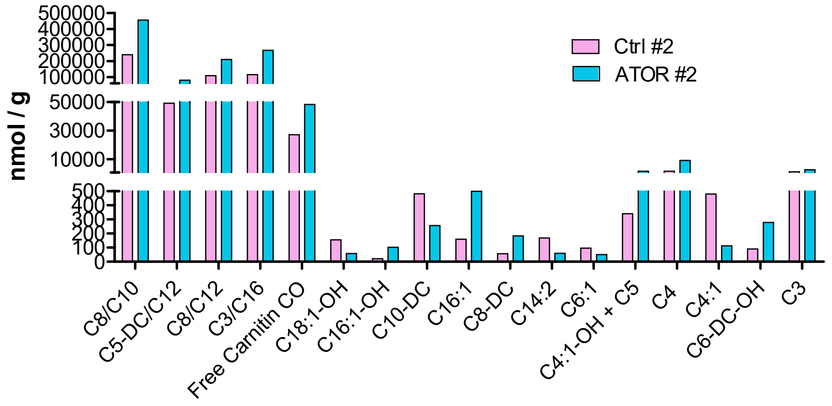

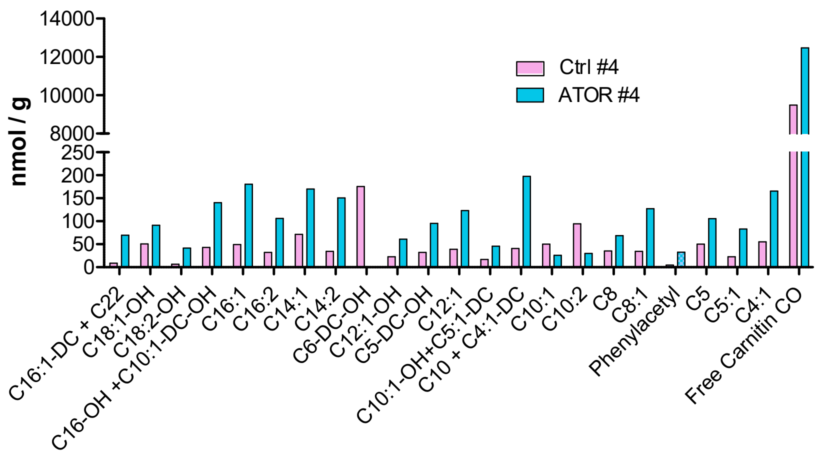

Supplementary Figure S8

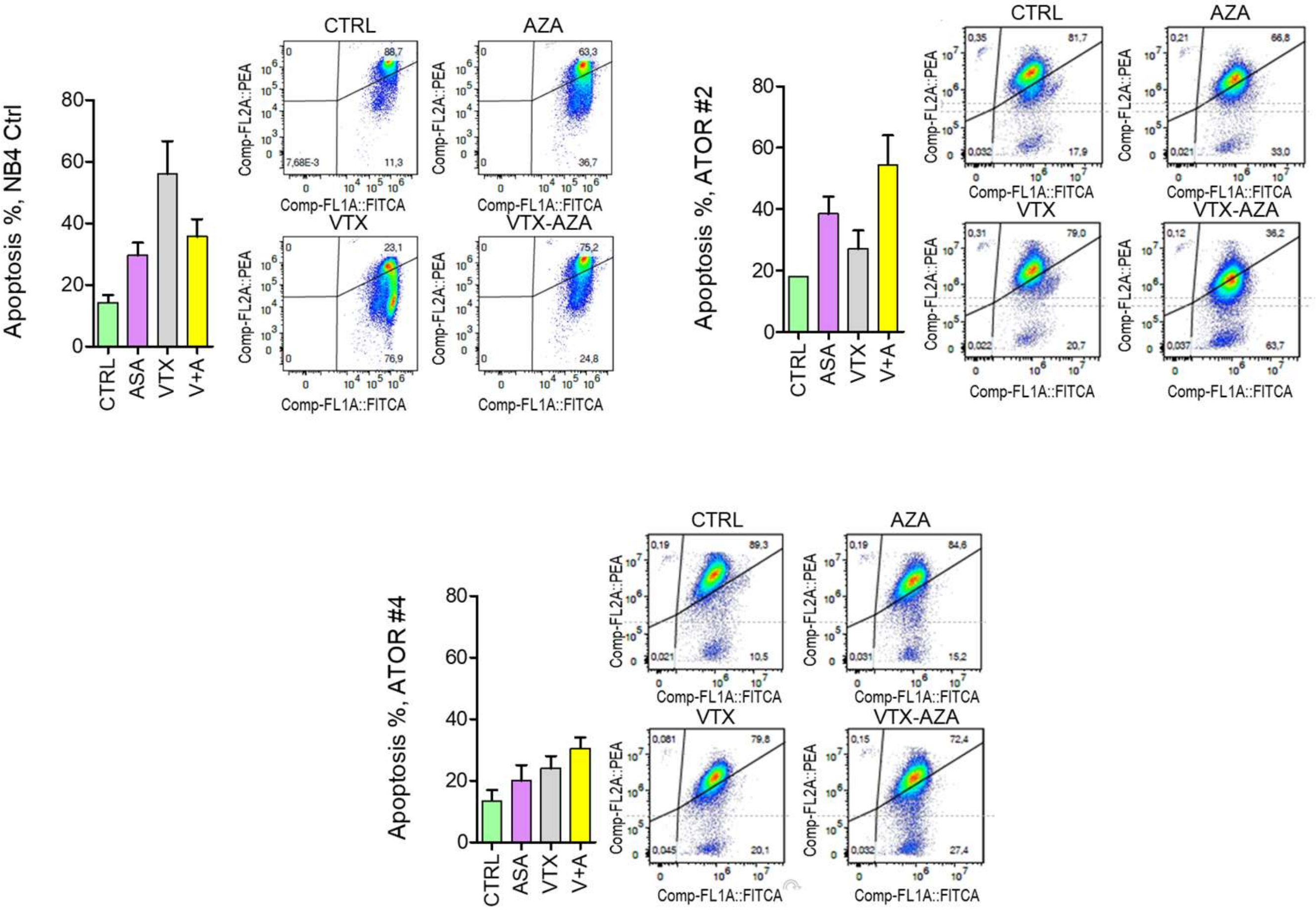

Supplementary Methods

Obtaining early progenitor precursors (EP/P)

Obtaining early progenitor precursors (EP/P), CD34+ cells were grown in serum substitute BIT 9500 (STEMCELL Technologies, Vancouver, Canada), a serum-free medium supplemented with IL-3 (1 unit/ml), GM-CSF (0.1 ng/ml) and saturating amounts of G-CSF (500 units/ml) to induce their differentiation into promyelocytes (Day 7) and granulocytes (Day 13). The morphologic and immunophenotypic characterization of the cells grown under these conditions was performed as previously described[1]

Cell lines and cell cultures

The APL-derived NB4 cell line carrying the t(15;17) translocation was purchased from DSMZ (Braunschweig, Germany). The two ATO resistant NB4 subclones (ATOR #2 and ATOR #4) were obtained by using a limiting dilution selection protocol. Briefly, NB4 cell clones were left untreated (paired controls) or treated with increasing concentrations of ATO (up to 1 μM) for 1 year, and resistant cells were selected as reported[2]. The human promonocytic U937 cell line carrying the PML::RARA sequence under the control of the zinc-inducible MT-1 promoter (PR9) or an empty MT-1 vector (MT)[3]. Cell lines were grown in RPMI medium (GIBCO-BRL, Grand Island, NY, USA) supplemented with 10% fetal bovine serum (FBS) (GIBCO-BRL), 20 mM Hepes, 100 μg/mL penicillin and 100 μg/mL streptomycin (GIBCO-BRL) at 37 °C in a humidified atmosphere containing 5% CO_2. Cells were tested regularly for mycoplasma contamination using a PCR kit (N-Garde EMK090020, Euroclone, Milan, Italy). MT and PR9 cells were treated with 100 μM $ZnSO_4$ for 24 hours. Arsenic trioxide (Sigma-Aldrich, Steinheim, Germany), was dissolved in water and added to the culture medium at 1 μM for 24 hours, as previously reported[4].

Cell viability assay

-ATP-based assay CellTiter-Glo® Viability Assay (Promega®, Madison, USA), cells were seeded at 1 x 10^4 cell per well into 96-well plates in triplicates and cultured for 72h with or without the treatments indicated in the text., according to manufacturer’s instructions.
-MTS assay A CellTiter 96® AQueous One Solution Cell Proliferation Assay. Cells were seeded in a 96-well plate at an initial density of 7 x 10^3 cell/well and treated for 72 h at 37 °C with the following compounds used alone or in combination: arsenic trioxide (ATO) at concentrations ranging from 0 to 1 μM; sulfo-N-succinimidyl oleate (SSO) at concentrations ranging from 0 to 100 μM; azacytidine (AZA), at concentrations ranging from 0 to 1000 mg/ml; venetoclax (VTX) at concentrations ranging from 0 to 300 nM. Subsequently, 5 l of the CellTiter 96® AQueous One Solution Cell Proliferation Assay (Promega; Madison, WI, USA) were added to each well for 4 h at RT. Cell absorbance was measured at 490 nm using a microplate reader (Thermo Scientific™ Varioskan™ Flash Multimode Reader; Waltham, MA, USA). Synergism analysis was performed using SynergyFinder tool (https://synergyfinder.fimm.fi/synergy/synfin_docs/).

Seahorse Assays

The mitochondrial and glycolytic functions were assessed with a Seahorse Bioscience XFe96 analayzer (Agilent Technologies, 102416-100). Extracellular flux assay kits, all from Agilent technologies, were used to measure basal oxygen consumption (OCR) and extracellular acidification rate (ECAR). The Agilent Seahorse XF Real-Time ATP Rate

Assay measures and quantifies, simultaneously, the rate of ATP (adenosine triphosphate) production from glycolysis and mitochondria in live cells. Oxidation Substrate Stress Test kit enables assessment, in live cells, of the oxidation of long chain fatty acids (LCFA), pyruvate and glutamine, the three primary mitochondrial substrates. Briefly, AML cells lines (6 x 10^4 cells/well), or primary APL blasts (2 x 10^5 cells/well were seeded in a Cell-Tak (Corning, 324240) coated 96-well XF96 well plate. Prior to analysis, the plate was incubated at 37°C without CO_2 for 1 hour. Analyses were performed at basal conditions and after injection of 2 μM oligomycin (Sigma-Aldrich, 871744), 1 μM FCCP (Sigma-Aldrich, C2920), 0.5 mM Antimycin A (Sigma-Aldrich, A8774), and 0.5 mM rotenone (Sigma-Aldrich, R8875) for the Cell Mito Stress Test and 2 μM Oligomycin and 50 mM 2-Deoxy-D-glucose (Sigma-Aldrich, D6134), for the glycolysis stress test. For Oxidation Substrate

Stress Test, specific inhibitors Etomoxir (ETO) (LCFA), UK5099 (Pyruvate) and BPTES (glutamine) were injected.

Analysis of amino acids and acylcarnitines

Amino acids (AA) were measured after precipitating proteins by adding 50 μl of 10% aqueous sulfosalicylic acid to 200 μl of medium. Samples were centrifuged for 8 min at 3600g and the supernatant was analyzed with a Biochrom 30 amino analyzer (Biochrom) using ion exchange chromatography and post-column ninhydrine derivatization.

Acylcarnitines were measured after butylation with a modified method for acylcarnitine measurement in newborn screening. Briefly, pellets were homogenized in 400 μl of PBS. After centrifugation at 13,000 rpm and 4 °C, for 15 minutes, the supernatant was used for measuring the metabolites. 50 μl of lysate was added to 450 μl acetonitrile and 200 μl methanol containing stable isotopically labeled acylcarnitines (Cambridge Isotope Laboratories, Inc.) as internal standard to denature proteins. After centrifuging the samples for 5 min at 3600 g, the supernatant was transferred into another vial, subsequently evaporated with a gentle nitrogen flow at 65 °C and butylated with 3 M butanolic hydro-chloric acid solution for 15 min at 65 °C. Remaining derivatization reagent was evaporated again with nitrogen and was resolved in 400 μl acetonitrile/water (80:20). 20 μl of the sample was injected into a LC-MS/MS system (Acquity UPLC connected to a Quattro Premier XE1, both Waters GmbH, Eschborn, Germany) via Flow Injection-MS/MS. Precursor-Scan of m/z 85 (common Fragment of acylcarnitines and free carnitine) was performed and acylcarnitines were quantified by relying on the stable isotopically labeled acylcarnitines where available or the stable isotopically labeled compound with the closest mass.

Analysis of TCA cycle intermediates

Pellets were homogenized in 70:30 acetonitrile:methanol (v/v) in the presence of 0.2 mM propane-1,2,3-tricarboxylic acid as an internal standard. The homogenate was centrifuged at 13,000 rpm and 4 °C, for 15 min. The supernatant was evaporated under nitrogen flow and derivatized by adding N-tert-butyldimethylsilyl-N-methyltrifluoroacetamide (MTBSTFA). After incubation at 80 °C for 60 minutes, the derivatized samples were measured by gas chromatography/ mass spectrometry using an Agilent 7890B GC equiped with an Agilent 5977A MSD (California, USA). The samples were injected into an Agilent CP-Sil 5 CB column (25 length, 0.25 diameter, 0.12 μm film thickness). The initial temperature was set to 50 °C for 1 minute, then it was increased to 200 °C at a rate of 4 °C/min, following a further increase at a rate of 20 °C/min until the final temperature of 270 °C was reached. The protein concentration was used for normalization.

Referencres

1. Careccia S, Mainardi S, Pelosi A, Gurtner A, Diverio D, Riccioni R, et al. A restricted signature of miRNAs distinguishes APL blasts from normal promyelocytes. Oncogene 2009 Nov 12; 28(45): 4034-4040.
2. Giansanti M, De Gabrieli A, Prete SP, Ottone T, Divona MD, Karimi T, et al. Poly(ADP-Ribose) Polymerase Inhibitors for Arsenic Trioxide-Resistant Acute Promyelocytic Leukemia: Synergistic In Vitro Antitumor Effects with Hypomethylating Agents or High-Dose Vitamin C. J Pharmacol Exp Ther 2021 Jun; 377(3): 385-397.
3. Piredda ML, Gaur G, Catalano G, Divona M, Banella C, Travaglini S, et al. PML/RARA inhibits expression of HSP90 and its target AKT. Br J Haematol 2019 03; 184(6): 937-948.
4. Noguera NI, Pelosi E, Angelini DF, Piredda ML, Guerrera G, Piras E, et al. High-dose ascorbate and arsenic trioxide selectively kill acute myeloid leukemia and acute promyelocytic leukemia blasts in vitro. Oncotarget 2017 May 16; 8(20): 32550-32565.
