# Supplementary material for: PML::RARα+ myeloid cells display metabolic alterations that can be targeted to treat resistant/relapse acute promyelocytic leukemias
Source: Leukemia. 2025 Sep 10;39(11):2708–20. doi: 10.1038/s41375-025-02738-9 (PMC12589136; doi:10.1038/s41375-025-02738-9)
Supplement: Supplementary file 2 — Supplementary Tables [file 41375_2025_2738_MOESM2_ESM.pdf]

Supplementary Table S1 Molecular and clinical characterization of AML primary blasts

	AGE	Molecular Biology	Diagnosis	Sanz Risk
1	NA	BCR3 isoform of *PML::RARα* ; *FLT3-D835+*	APL	High
2	23	BCR2 isoform of *PML::RARα*	APL	High
3	44	BCR3 isoform of *PML::RARα*	APL	Intermediate
4	53	BCR1 isoform of *PML::RARα*	APL	High
5	42	BCR1 isoform of *PML::RARα*	APL	NA
6	65	BCR1 isoform of *PML::RARα*	APL	Low
7	4	BCR3 isoform of P*ML::RARα*	APL	High
8	76	BCR1 isoform of *PML::RARα*	APL	High
9	9	BCR3 isoform of P*ML::RARα*	APL	High
10	42	BCR1 isoform of *PML::RARα*	APL	Low
11	24	BCR2 isoform of *PML::RARα*	APL	High
12	52	BCR1 isoform of *PML::RARα*	APL	Intermediate
13	85	BCR3 isoform of P*ML::RARα*	APL	High
14	9	BCR3 isoform of P*ML::RARα*	APL	High
15	85	BCR3 isoform of P*ML::RARα*	APL	High
16	42	*RUNX1::RUNX1T1*	AML	-
17	23	Negative	AML	-
18	62	*NPM mut*	AML	-
19	64	*FLT3-ITD+ / NPM mut*	AML	-
20	68	*FLT3-ITD+*	AML	-
21	58	Negative	AML	-
22	69	Negative	AML	-
23	68	Negative	AML	-
24	71	Negative	AML	-
25	60	*FLT3-ITD+*	AML	-
26	39	Negative	AML	-
27	69	*NPM mut*	AML	-
28	63	Negative	AML	-
29	73	*CBFB::MYH11*	AML	-
30	65	Negative	AML	-
31	85	*FLT3-ITD+*	AML	-
32	75	Negative	AML	-
33	56	*FLT3-ITD+; NPM mut*	AML	-
34	39	Negative	AML	-
35	48	Negative	AML	-

Panel: *RUNX1::RUNX1T1* ; *BCR::ABL1* ; *CBFB::MYH11* ; *FLT3-ITD* ; *FLT3 D835* ; *NPM* ; DEK::NUP214; N.A: not available

Supplementary Tables

Supplementary Table S2 Glycolysis and Mitochondrial respiration values in APL blasts and normal hematopoietic cells

		APL	EP/P (N7)	EP/P (N13)	NBM	p-value (AML vs EP/P (N7)
**Glycolysis** ECAR(mpH/min/105 Cells)	**Basal**	11±7	37±6	18±4	3±0	0.001
	**Capacity**	15±9	41±10	28±7	4±1	0.003
	**Reserve**	5±5	10±3	11±5	0.5±0.7	0.16
**Mitochondrial Respiration** OCR(pmol/min/105 Cells)	**Basal**	22±9	50±12	24±16	9±1	0.003
	**SRC**	114±58	153±54	56±56	23±4	0.05
	**PL**	115±43	10±2	5±1	0.5±0.7	n.s.
	**ATP**	18±9	41±10	20±16	8±1	0.01

ECAR (extra cellular acidification rate); OCR (oxygen consume rate); EP/P (early progenitors/precursors; N7 (at day 7); N13 (at day 13); NBM (normal bone marrow); SRC (spare reserve capacity); PL (Proton Leak). Values represent the mean ± SD. Statistical analysis was performed using the Student's t-test.

Supplementary Table S3 Mitochondrial respiration and Glycolysis and values in MT (Ctrl) and PR9 (PML::RARA+) cells

		MT+Zn	PR9+Zn	p-value
**Glycolysis** ECAR(pmol/min/105 Cells)	**Basal**	827±61	396±23	<0.0001
	**Compensatory**	1031±115	667±79	<0.0001
**Mitochondrial Respiration** OCR(pmol/min/105 Cells)	**Basal**	99±26	129±18	0.05
	**Max. Resp.**	201±15	234±43	0.1
	**SRC**	26±2	30±2	0.01
	**PL**	62±14	94±11	0.002
	**ATP**	113±26	110±47	0.9

ECAR (extra cellular acidification rate); OCR (oxygen consume rate); Max. Resp. (maximal respiration); SRC (spare respiratory capacity). Values represent the mean ± SD. Statistical analysis was performed using the Student's t-test.

Supplementary Table S4 List of reagents

Reagent	Function	Source	Identifier
Arsenic Trioxide (ATO)	PML::RARα degradation	Sigma-Aldrich, Germany	# 31,138-3
Inhibitor VIII	AKT inhibitor	Enzo life sciences, Lause, Switzerland	ENZ-CHM125
Venetoclax (VTX)	Bcl-2 inhibitor	SelleckChem, Köln, Germany	# S8048
5-Azacitidine (AZA)	DNA metiltrasferasa inhibitor	SelleckChem, Köln, Germany	# S1782
Sulfo-N-succinimidyl Oleate sodium (SSO)	CD36 inhibitor	Sigma-Aldrich, Germany	SML2148-5MG
Perhexiline	Carnitine Palmitoyl Transferase (CPT-1) inhibitor	Sigma-Aldrich, Germany	# SML0120
Zinc Sulfate	Inducer	Sigma-Aldrich, Steinheim, Germany	# Z0251
2-Deoxyglucosio	glycolysis inhibitor	Sigma-Aldrich, Steinheim, Germany	D8375-5G
Etomoxir (ETO)	Carnitine Palmitoyl Transferase (CPT-1) inhibitor	Sigma-Aldrich, Steinheim, Germany	236020
BPTES	Glutaminase inhibitor	Sigma-Aldrich, Steinheim, Germany	SML0601
UK5099	MCT inhibitor (mitochondrial pyruvate carrier)	Sigma-Aldrich, Steinheim, Germany	PZ0160
Oligomycin	inhibitor of ATP synthase (blocks Complex V)	Sigma-Aldrich, Steinheim, Germany	O4876
Carbonyl cyanide p-(trifluoromethoxy) phenylhydrazone (FCCP)	Uncoupler of mitochondrial oxidative phosphorylation	Sigma-Aldrich, Steinheim, Germany	C2920
Poly-L-lysine solution	Adhesive subbing solution	Sigma-Aldrich, Steinheim, Germany	P4832
Antimycin A	Blocks mitochondrial electron transport chain complex III	Sigma-Aldrich, Steinheim, Germany	A8674
Rotenone	NADH-CoQ reductase inhibitor (blocks Complex I)	Sigma-Aldrich, Steinheim, Germany	R8875

Supplementary Table S5 List of Primary Antibodies

Antibody	Source	Identifier
PARP1	Cell Signaling, Danvers, MA, USA	#9541
p-AKT308	Cell Signaling, Danvers, MA, USA	#2965
AKT	Cell Signaling, Danvers, MA, USA	#9272
SLC22A16 (CT2)	Novus Biological, Toronto, ON, Canada	NBP1-85410
β-Actine	Sigma , Deutschland, GmbH	# 3700
MCL-1	Cell Signaling, Danvers, MA, USA	# 5453
BCL-XL	Cell Signaling, Danvers, MA, USA	54H6
BCL2	Cell Signaling, Danvers, MA, USA	# 15071
Caspase 3	Cell Signaling, Danvers, MA, USA	# 9664
HK2	Proteintech (USA)	22029-1-AP
PFKP	Cell Signaling, Danvers, MA, USA	# 8164
PKM2	Cell Signaling, Danvers, MA, USA	# 3190
RARA	Cell Signaling, Danvers, MA, USA	# 62294
Tubulin	Sigma, Deutschland, GmbH	T 5168
GAPDH	Nobus Biologicals, Toronto, ON, Canada	NB300-221SS
PDHA1	RayBiotech, Georgia, GA, USA	144-01895-50

Supplementary Table S6 Primers used

GENE	FORWARD	REVERSE
PDHA1	5'- aggcgatttcattcctggg -3'	5'- ctcatactgtgtccgtggtaac -3'
SLC22A16 (CT2)	5'- cggcgtttgcagttgattat -3'	5'- tccgagacttcatgccaatg -3'
ABL	5'- tggagataacactctaagcataactaaaggt-3'	5'- gatgtagttgcttgggaccca -3'
